# Modelo preditivo para complicações em artroplastia total do quadril: Desenvolvimento de uma ferramenta clínica

**DOI:** 10.1055/s-0046-1824727

**Published:** 2026-10-06

**Authors:** Andrea Moreira Candido, Conrado Torres Laett, Alanna Martins Soares de Palma, Lourenço Pinto Peixoto, Sidnei Cavalcante da Silva

**Affiliations:** 1 Programa de Pós-Graduação em Ciências Aplicadas ao Sistema Musculoesquelético, Divisão de Ensino e Pesquisa (DIENP), Instituto Nacional de Traumatologia e Ortopedia (INTO), Rio de Janeiro, RJ, Brasil; 2 Laboratório de Pesquisa Neuromuscular, Divisão de Pesquisa, Instituto Nacional de Traumatologia e Ortopedia (INTO), Rio de Janeiro, RJ, Brasil; 3 Centro de Cirurgia do Quadril, Divisão de Traumatologia e Ortopedia (DITRO), Instituto Nacional de Traumatologia e Ortopedia (INTO), Rio de Janeiro, RJ, Brasil; 4 Programa de Pós-Graduação em Ciências da Reabilitação, Centro Universitário Augusto Motta (UNISUAM), Rio de Janeiro, RJ, Brasil; 5 Laboratório de Pesquisa em Fisiologia do Exercício, Divisão de Pesquisa, Instituto Nacional de Traumatologia e Ortopedia (INTO), Rio de Janeiro, RJ, Brasil

**Keywords:** artroplastia de quadril, fatores prognósticos, fatores de risco, modelos preditivos de aprendizagem, arthroplasty, replacement, hip, predictive learning models, prognostic Factor, risk factors

## Abstract

**Objetivo:**

Desenvolver um modelo preditivo para estimar complicações até 30 dias após artroplastia total de quadril (ATQ), com base em variáveis clínicas e funcionais, visando à criação de uma ferramenta prática para uso clínico.

**Métodos:**

Estudo de coorte prospectivo realizado em hospital público de referência, com pacientes submetidos à ATQ primária e eletiva entre novembro de 2021 e abril de 2022. A amostra incluiu 122 pacientes (média de idade: 56 anos, 59,8% homens). Foram avaliadas variáveis clínicas, sociodemográficas e de capacidade funcional, estimada pelo Duke Activity Status Index (DASI). Os desfechos incluíram intercorrências clínicas, reinternação precoce, internação prolongada (> 6 dias) e permanência em UTI por mais de um dia. Utilizou-se modelo de regressão logística, com desempenho avaliado pela estatística C (AUC), incorporado a uma ferramenta online.

**Resultados:**

As comorbidades mais prevalentes foram hipertensão (61%), diabetes (23%) e dislipidemia (15%). Complicações em até 30 dias ocorreram em 13,1% dos pacientes, sendo infecção da ferida operatória e anemia os eventos mais comuns. Dislipidemia (RC: 4,70; *p* = 0,010), sedentarismo (RC: 4,71; *p* = 0,026), ASA elevado (RC: 3,67; *p* = 0,035) e capacidade funcional < 4 equivalentes metabólicos da tarefa (METs, do inglês metabolic equivalents of task) (RC: 4,08; *p* = 0,040) foram fatores de risco significativos. A ferramenta está disponível em https://upneuro.shinyapps.io/atq-pred .

**Conclusão:**

Dislipidemia, sedentarismo, baixa capacidade funcional e ASA elevada associaram-se ao maior risco de complicações pós-ATQ. A avaliação pré-operatória deve incluir aspectos funcionais e cardiovasculares. O modelo mostrou boa acurácia, mas novos estudos são necessários para validar sua aplicação clínica.

**Nível II – Evidência de nível moderado.:**

Trata-se de um estudo de coorte prospectivo, sem grupo controle, voltado à identificação de fatores prognósticos para complicações em artroplastia total de quadril.

## Abstract (en)

**Objective:**

To develop a predictive model to estimate complications up to 30 days after total hip arthroplasty (THA), based on clinical and functional variables, aiming at the creation of a practical tool for clinical use.

**Methods:**

Prospective cohort study conducted in a public referral hospital, with patients undergoing primary and elective THA between November 2021 and April 2022. The sample included 122 patients (mean age: 56 years, 59.8% men). Clinical, sociodemographic, and functional capacity variables were evaluated and estimated by the Duke Activity Status Index (DASI). Outcomes included clinical complications, early readmission, prolonged hospitalization (> 6 days), and ICU stay for more than one day. A logistic regression model was used, with performance evaluated by the C-statistic (AUC), and the model was incorporated into an online tool.

**Results:**

The most prevalent comorbidities were hypertension (61%), diabetes (23%), and dyslipidemia (15%). Complications within 30 days occurred in 13.1% of patients, with surgical wound infection and anemia being the most common events. Dyslipidemia (OR: 4.70; *p* = 0.010), sedentary lifestyle (OR: 4.71; *p* = 0.026), high ASA classification (OR: 3.67; *p* = 0.035), and functional capacity < 4 metabolic equivalents of task (METs) (OR: 4.08; *p* = 0.040) were significant risk factors. The tool is available at https://upneuro.shinyapps.io/atq-pred .

**Conclusion:**

Dyslipidemia, sedentary lifestyle, low functional capacity, and high ASA classification were associated with a higher risk of post-THA complications. The preoperative evaluation should include functional and cardiovascular aspects. The model showed good accuracy, and further studies are needed to validate its clinical application.

**Level II – Moderate level evidence.:**

This is a prospective cohort study without a control group aimed at identifying prognostic factors for complications after total hip arthroplasty.

## Introdução

A artroplastia total de quadril (ATQ) é o procedimento cirúrgico de substituição da articulação do quadril por prótese, visando restabelecer a função articular e aliviar a dor. É uma das cirurgias eletivas mais realizadas e bem-sucedidas no mundo, com mais de um milhão de procedimentos ao ano, 1 2 sendo considerada por muitos como a cirurgia ortopédica do século devido aos bons resultados clínicos. 3

Apesar dos avanços na assistência perioperatória, complicações no período pós-operatório ainda são frequentes, com a morbidade em 30 dias pós ATQ variando entre 5 e 20%. 4 5 6

A estratificação de risco pré-operatório permite identificar fatores potencialmente modificáveis, otimizar condutas clínicas, orientar estratégias no intra- e pós-operatório e auxiliar na gestão de recursos. 7 8 9 Além disso, modelos preditivos contribuem para a tomada de decisão compartilhada entre equipe médica e paciente quanto à realização do procedimento. 10

Diversos índices ou escores de risco são utilizados para prever complicações em cirurgias não-cardíacas, como a classificação da Sociedade Americana de Anestesiologia (ASA), o índice de risco cardíaco revisado (RCRI) e o escore do estudo multicêntrico de avaliação perioperatória (EMAPO). 11 12 13 Para aprimorar essa predição em cirurgias ortopédicas, Waterman et al. 14 propuseram o índice de risco cardíaco para artroplastia articular total (CRI-TJA), baseado em dados da população dos EUA. 15

Dentre os principais fatores associados à morbimortalidade após ATQ estão: idade superior a 75 anos, escore ASA ≥ 3, sexo masculino e comorbidades, como doença cardiovascular, hipertensão arterial sistêmica (HAS), obesidade e doença cerebrovascular. 14 16 Embora estudos apontem a baixa capacidade funcional como fator de risco relevante para eventos adversos em cirurgias não cardíacas, 17 18 a limitação imposta pela doença ortopédica dificulta sua avaliação objetiva em candidatos à ATQ. Nestes casos, instrumentos como o índice de aptidão física de Duke (DASI, do inglês Duke Activity Status Index) 19 podem representar uma alternativa viável para avaliar a capacidade funcional de forma indireta e padronizada. De acordo com Wijeysundera et al., 20 o DASI foi associado a maior risco de complicações ou morte em 30 dias após cirurgias não cardíacas de grande porte.

Na população brasileira, a identificação de fatores de risco e o desenvolvimento de ferramenta de predição de complicações após ATQ ainda são campos a serem explorados, sendo este o objetivo do presente estudo.

## Materiais e Métodos

Pacientes submetidos a ATQ primária eletiva em nossa instituição, no período de 16 de novembro de 2021 a 16 de abril de 2022 foram convidados a participar de coorte prospectiva com seguimento de 30 dias para avaliação de eventos adversos. Foram incluídos pacientes com mais de 18 anos, enquanto aqueles com cirurgia cancelada ou incapacidade de compreender os questionários utilizados foram excluídos. Os dados obtidos foram utilizados para desenvolvimento de modelo prognóstico. O estudo foi aprovado previamente pelo comitê de ética em pesquisa com seres humanos da instituição (CAAE: 36510920.8.0000.5273) e todos os participantes assinaram termo de consentimento livre e esclarecido.

A capacidade física dos pacientes foi avaliada por meio do DASI, traduzido e validado para português, aplicado no formato de entrevista pelo pesquisador responsável. 21 O questionário é composto por 12 itens com pesos variados, cuja soma é utilizada para estimativa do consumo máximo de oxigênio por minuto (VO _2máx_ ) através da equação descrita por Hlatky et al. 22 Além disso, o VO _2máx_ foi expresso em equivalentes metabólicos da tarefa (MET, do inglês *metabolic equivalent of task* ), sendo 1 MET: 3,5 L/min. Para minimizar interferência com o procedimento usual de internação, dados demográficos, exames laboratoriais e classificação ASA foram obtidos do prontuário. Os dados do paciente foram utilizados para estratificação de risco pré-operatório de acordo com o RCRI, CRI-TJA e EMAPO.

Os pacientes foram acompanhados por análise do prontuário do início da cirurgia até o dia 30 pós-operatório. Foram considerados eventos adversos as complicações moderadas ou graves de acordo com a classificação de Clavien-Dindo adaptada por Wijeysundera et al. 23 Complicações moderadas e graves são, respectivamente, quadros que requerem tratamento, mas não causam danos permanentes; e quadros que levam a risco potencial de morte e/ou requerem internação em unidade de terapia intensiva.

Dados numéricos contínuos, discretos e dicotômicos estão reportados como média ± desvio padrão (DP), mediana (intervalo interquartílico [IIQ]) e ocorrência absoluta (percentual). A associação entre os fatores e eventos registrados foi avaliada por razão de chance (RC) obtida por regressão logística univariada, sendo aqueles com valor de *p* < 0,10 no teste de Wald incluídos em modelo logístico multivariado. Foram considerados fatores de risco independentes aqueles com *p* < 0,05 no teste de Wald no modelo univariado.

O desempenho do modelo de regressão logística e dos escores de estratificação de risco foram avaliados pela área sob a curva *receiver operating characteristic* (ROC, estatística C) e comparados pelo método de DeLong. O modelo desenvolvido foi incluído em ferramenta online de cálculo de risco desenvolvida para o projeto (UPNEURO-INTO, https://upneuro.shinyapps.io/atq-pred ). Todos os procedimentos foram desenvolvidos em rotinas em Python 3.10 (Python Software Foundation).

## Resultados

Ao todo, 122 pacientes participaram do estudo, sendo a osteoartrite (OA) primária a indicação em 86 casos (70,4%) e OA secundária em 36 (29,6%). As causas secundárias de OA foram trauma, osteonecrose, displasia do desenvolvimento do quadril e sequela de artrite séptica. Dentre eles, 16 (13,1%) passaram por eventos adversos em 30 dias de seguimento, tendo ocorrido complicações durante a internação inicial em 10 pacientes (8,2%), além de 6 reinternações (4,9%). Somado a isso, 2 pacientes apresentaram múltiplas intercorrências, um com anemia com necessidade de hemotransfusão e infecção da ferida operatória, outro com anemia com necessidade de hemotransfusão, taquiarritmia e crise hipertensiva.

As complicações observadas isoladamente foram em 3 casos anemia com necessidade de hemotransfusão, assim como 2 casos de luxações da prótese, 1 caso de infecção respiratória aguda (IRA), 1 de taquiarritmia e 1 de exacerbação de doença pulmonar obstrutiva crônica (DPOC). Em 4 casos, a causa da reinternação foi infecção de ferida operatória, em um caso infecção periprotética e, no restante, suspeita de trombose venosa profunda (TVP).

Foi possível identificar fatores de risco para os eventos adversos, apresentados em detalhes junto às características da amostra na Tabela 1 .

**Tabela 1:** Características dos participantes e fatores de risco

	Total	Pacientes acometidos	Pacientes não-acometidos	Valores de *p*	RC	IC 95%
**Sexo masculino**	60% (73)	56% (9)	60% (64)	0,754	0,84	0,29–2,44
** IMC (kg/m ^2^ ) **	29,01 ± 6,0	28,8 ± 7,0	29,0 ± 5,8	0,864	0,99	0,91–1,09
** < 25**	25% (31)	31% (5)	25% (26)	0,566	1,40	0,44–4,40
** 25–29,9**	39% (47)	31% (5)	40% (42)	0,523	0,69	0,22–2,14
** 30–34,9**	20% (25)	19% (3)	21% (22)	0,853	0,88	0,23–3,37
** 35–39,9**	9% (11)	12% (2)	8% (9)	0,604	1,54	0,30–7,87
** > 40**	7% (8)	6% (1)	7% (7)	0,958	0,94	0,11–8,21
**Idade (anos)**	56,4 ± 12,9	58,1 ± 13,2	56,1 ± 12,9	0,572	1,01	0,97–1,06
** < 60**	56% (68)	50% (8)	57% (60)	0,621	0,77	0,27–2,20
** 60–79**	40% (49)	38% (6)	41% (43)	0,816	0,88	0,30–2,60
** ≥ 80**	4% (5)	12% (2)	3% (3)	0,096	4,90	0,75–31,96
**Dislipidemia**	**15% (18)**	**38% (6)**	**11% (12)**	**0,010**	**4,70**	**1,45–15,25**
**Hipertensão**	61% (75)	81% (13)	58% (62)	0,094	3,08	0,83–11,44
**Diabetes**	23% (28)	31% (5)	22% (23)	0,400	1,64	0,52–5,20
**Sedentarismo**	**9% (11)**	**25% (4)**	**7% (7)**	**0,026**	**4,71**	**1,20–18,49**
**Tabagismo**	12% (15)	19% (3)	11% (12)	0,404	1,81	0,45–7,27
**METs (DASI)**	5,6 ± 1,2	5,1 ± 1,3	5,6 ± 1,2	0,141	0,69	0,43–1,13
** < 4**	10% (12)	**25% (4)**	**8% (8)**	**0,040**	**4,08**	**1,07–15,62**
** 4–6,9**	76% (93)	69% (11)	77% (82)	0,453	0,64	0,20–2,04
** > 7**	14% (17)	6% (1)	15% (16)	0,358	0,38	0,05–3,04
**ASA**		**2,1 ± 0,5**	**1,8 ± 0,5**	**0,035**	**3,67**	**1,09–12,33**
** 1**	18,0% (22)	6% (1)	20% (21)	0,217	0,27	0,03–2,16
** 2**	75,4% (92)	75% (12)	75% (80)	0,967	0,97	0,29–3,29
** 3**	6,6% (8)	19% (3)	5% (5)	0,051	4,66	1,–21,82
** 4**	0% (0)	0% (0)	0% (0)	−	−	−

**Abreviações:** ASA, Sistema de Classificação do Estado Físico da Sociedade Americana de Anestesiologistas; ATQ, artroplastia total de quadril; DASI, Duke activity status index; IC 95%: intervalo de confiança de 95%; IMC, índice de massa corporal; MET, equivalente metabólico da tarefa; RC, razão de chances.

**Notas:** Os dados dicotômicos estão apresentados como percentual de ocorrência (número) e os dados numéricos como média ± desvio padrão. Comorbidades com prevalência menor que 15% não foram relatadas e nenhuma foi identificada como fator de risco. Valor de *p* referente ao teste de Wald.

Outras comorbidades observadas foram: doença renal crônica (5%), hipotireoidismo (4%), insuficiência venosa (2%) e asma (2%).

A classificação de acordo com os escores de risco avaliados está apresentado na Tabela 2 , com exceção do ASA, avaliado como fator de risco e potencial componente da ferramenta desenvolvida, disponível na Tabela 1 .

**Tabela 2:** Escores de risco

RCRI		CRI-TJA		EMAPO	
**1**	91,0% (111)	**0**	37,7% (46)	**0**	0% (0)
**2**	8,2% (10)	**1**	59,0% (72)	**1–5**	74,6% (91)
**4**	0,8% (1)	**2**	3,3% (4)	**6–10**	23,7% (29)
**5**	0% (0)	**3**	0% (0)	**11–15**	1,63% (2)
				**>15**	0% (0)

**Abreviações:** CRI-TJA, índice de risco cardíaco para artroplastia articular total; EMAPO, estudo multicêntrico de avaliação perioperatória; RCRI, índice de risco cardíaco revisado.

**Nota:** Os dados dicotômicos estão apresentados como percentual de ocorrência (número).

O modelo desenvolvido corresponde a equação Logit (p) = -8,89 + oct*1,76 + disl*1,47 + hip*0,05 + sed*1,65 + DASI4*1,60 + ASA*1,02 , onde *p* é a probabilidade estimada de ocorrência de eventos adversos, *oct* indica se o paciente é octogenário, *disl* se dislipidêmico, *hip* se hipertenso, *sed* se sedentário, *DASI4* se menos de 4 METs estimados pelo DASI, e *ASA* é o valor numérico deste escore de risco.

O limiar ótimo identificado para o modelo foi de 0,20. O modelo apresentou desempenho superior ao ASA ( *p* = 0,002), RCRI ( *p* = 0,007), CRI ( *p* = 0,002) e EMAPO ( *p* < 0,001), como apresentado na Fig. 1 .

**Fig. 1:**
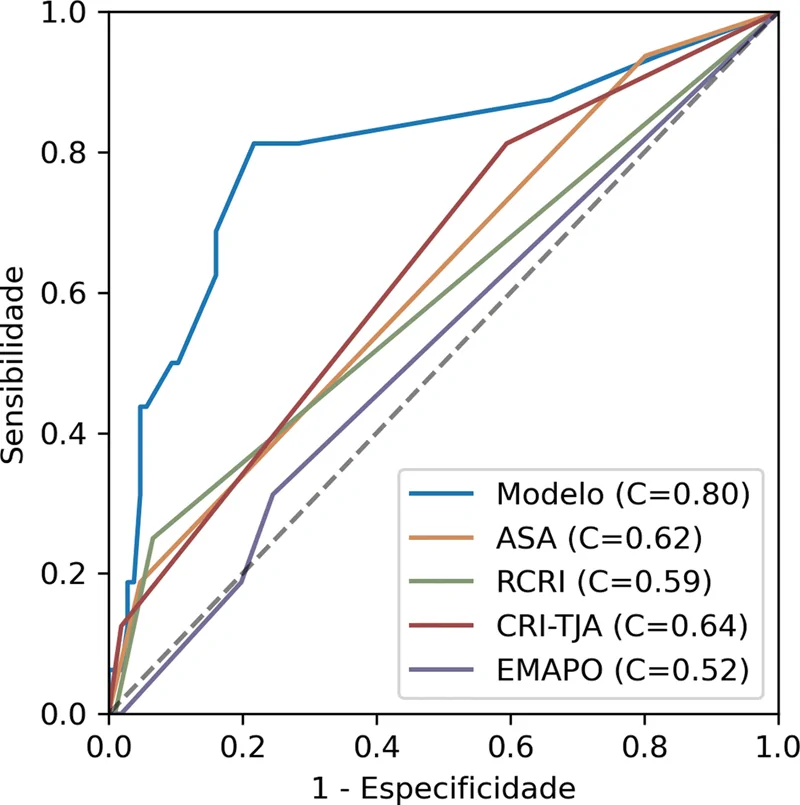
Desempenho do modelo desenvolvido e dos escores de risco avaliados, apresentado pela curva *receiver operating characteristics* (ROC) e quantificado pela área sob a ROC (estatística C). **Abreviações:** ASA, sistema de classificação do estado físico da American Society of Anesthesiologists (Sociedade Americana de Anestesiologistas); CRI-TJA, índice de risco cardíaco para artroplastia articular total; EMAPO, estudo multicêntrico de avaliação perioperatória; RCRI, índice de risco cardíaco revisado; ROC, receiver operating characteristics.

Para utilização da ferramenta UPNEURO-INTO o profissional deve acessar o link através de um computador ou dispositivo móvel e informar os dados do paciente. O DASI é apresentado no painel esquerdo da tela, podendo ser preenchido diretamente no formato de entrevista ou por registro prévio. O resultado (expresso em METS) é apresentado em tempo real no espaço abaixo do questionário. No painel direito superior da tela devem ser preenchidas as características clínicas no momento da internação através das caixas de seleção e o nível ASA indicado no menu logo abaixo. A estimativa de probabilidade de eventos adversos será atualizada em tempo real durante a inserção dos dados e apresentada no indicador localizado no painel inferior direito.

## Discussão

Os resultados deste estudo permitiram a identificação de fatores de risco associados a complicações ou reinternações em pacientes submetidos a ATQ primária eletiva. Entre esses fatores, destacam-se a dislipidemia, o sedentarismo, a capacidade funcional inferior a 4 METs estimada pelo DASI e classificação ASA progressiva. A partir desses achados, foi possível elaborar uma ferramenta de estimativa de risco de eventos adversos, com desempenho superior aos demais escores testados.

A amostra apresentou média de idade inferior a esperada, além do predomínio do sexo masculino, contrastando com dados anteriores que indicam maior número de mulheres com mais de 65 anos submetidas a ATQ. 1 24 A elevada proporção de casos de osteoartrite secundária (25%), incluindo etiologias como trauma, osteonecrose e displasia do desenvolvimento do quadril, pode contribuir para esse perfil etário e demográfico distinto. Considerando-se que a prevalência de doenças displasicas é maior entre homens com menos de 50 anos, 1 a elevada proporção de casos de osteoartrite secundária (25%) pode explicar esse perfil atípico.

Cerca de 75% dos pacientes apresentavam sobrepeso ou obesidade, em consonância com a literatura 25 e com o reconhecimento do sobrepeso e obesidade 26 como fatores de risco para OA. 1 As comorbidades mais frequentes foram HAS, diabetes mellitus (DM) e dislipidemia, com prevalências superiores às descritas em outros estudos. 14 Esses achados são compatíveis com o fenótipo de OA associado à síndrome metabólica, 27 marcado por inflamação crônica de baixo grau, embora ainda não haja consenso quanto a uma relação causal. 28

O alto percentual de fatores de risco para doença cardiovascular, como HAS, DM, dislipidemia, tabagismo e sedentarismo, reforça a importância de uma avaliação pré-operatória cuidadosa.

Poucos estudos ortopédicos avaliaram a capacidade funcional através do DASI, embora esse escore seja considerado útil em indivíduos com limitação de locomoção. 29 O valor médio (5,5 METs) obtido para capacidade funcional no estudo é semelhante ao mensurado em teste cardiopulmonar de exercício (TCPE) em pacientes com OA de quadril, 30 o que valida o DASI como ferramenta viável nessa população.

A maioria dos pacientes apresentou capacidade funcional moderada e 10% deles tiveram estimativa menor que 4 METs, valor inferior ao observado em populações cirúrgicas distintas, 23 o que pode ser atribuído à limitação imposta pela OA, mais do que a um déficit cardiopulmonar.

A maioria dos pacientes foi classificada com baixa pontuação nos escores de risco: ASA 2, RCRI com 1 ponto, CRI-TJA indicando pontuação mínima para 40% da amostra e o EMAPO classificando 75% como baixo risco. A escolha destes índices para comparação com a ferramenta de estudo se deve a ampla utilização do ASA e do RCRI, pela especificidade do CRI-TJA para ATQ e pelo EMAPO ser um escore de risco desenvolvido com dados da população brasileira.

A taxa de eventos adversos (13,1%) é compatível com a literatura. 5 6 Não houve óbitos, o que pode indicar alto padrão assistencial, mas também pode estar relacionado ao tamanho reduzido da amostra. As complicações mais frequentes foram infecção local e anemia com necessidade de hemotransfusão, seguidas por taquiarritmias, luxação protética, crises hipertensivas, insuficiência renal aguda, suspeita de TVP e descompensação de DPOC – eventos bem documentados após ATQ. 16

As reinternações ocorreram em percentual semelhante ao descrito por Roger et al., 31 e superior ao relatado por Sørensen et al., 32 sendo majoritariamente motivadas por infecção e suspeita de TVP, em concordância com achados prévios. 16

Dislipidemia foi identificada como um dos fatores de risco isolado para complicações, achado inédito em estudos ortopédicos, embora síndrome metabólica já tenha sido associada a complicações e a reinternação em 30 dias após ATQ e artroplastia total de joelho. 33 A presença de HAS mostrou tendência a triplicar o risco de eventos adversos ou reinternações, semelhante ao observado por Waterman et al., 14 enquanto esse risco parece aumentar em cinco vezes em pacientes com 80 anos ou mais, embora o tamanho amostral possa ter limitado a significância estatística.

O sedentarismo e capacidade funcional inferior a 4 METs foram associados a aumento superior a quatro vezes na chance de eventos adversos. Estudos prévios já sugeriam essa relação por critérios subjetivos, como subir escadas, 17 34 ou por medidas objetivas como o consumo de oxigênio em TCPE. 35 O DASI já demonstrou valor preditivo em cirurgias não cardíacas, 23 36 sugerindo sua aplicação em avaliações pré-operatórias. Mesmo a autodeclaração de sedentarismo se mostrou útil como indicador de risco.

A identificação de pacientes com capacidade funcional inferior a 4 METs permite traçar estratégias de maior vigilância e intervenções específicas, como programas de exercícios prévios à cirurgia. Já se demonstrou redução no tempo de internação hospitalar com intervenções físicas pré-operatórias, 37 e ferramentas tecnológicas podem facilitar a adesão mesmo à distância. 38

A classificação ASA também se correlacionou a eventos adversos, corroborando estudos anteriores que relatam maiores taxas de complicações em pacientes com ASA ≥ 3. 6

Com base nos fatores identificados, foi proposto um modelo preditivo por regressão logística, eficaz na estimativa do risco de eventos adversos pós ATQ. A simplicidade dos dados utilizados torna a ferramenta aplicável na prática clínica. O modelo desenvolvido teve desempenho superior aos escores tradicionais analisados, demonstrando maior sensibilidade e especificidade na predição de complicações ou reinternações.

Este estudo apresenta algumas limitações. Primeiro, o DASI foi aplicado na forma de entrevista, e a capacidade cardiorrespiratória não foi mensurada pelo TCPE, que é outro padrão para essa avaliação. Também não foram incluídos parâmetros laboratoriais, como hemoglobina e creatinina, que poderiam representar fatores de risco adicionais. A dislipidemia foi identificada apenas por autorrelato ou uso de estatinas, sem confirmação laboratorial que permitisse detectar casos desconhecidos ou avaliar o controle do tratamento.

A análise por tipo específico de complicação (cardíacas, clínicas não cardíacas –ex.: trombose venosa profunda e insuficiência renal aguda – e cirúrgicas) não foi possível devido ao número reduzido de eventos e ao tamanho da amostra. Da mesma forma, a avaliação de lesão miocárdica exigiria exames como eletrocardiograma e dosagem de troponina, que não foram realizados.

Além disso, como se trata de um estudo piloto o modelo de predição ainda não passou por validação externa, etapa prevista para fases posteriores da pesquisa.

Por outro lado, o estudo apresenta pontos fortes: é prospectivo, pioneiro na utilização do DASI no pré-operatório de ATQ, além de propor um modelo de predição de risco específico para essa população. A criação de um protótipo computacional também destaca a aplicação clínica da proposta.

Apesar das limitações, os resultados abrem novas perspectivas para a estimativa de risco em ATQ. Estudos com amostras maiores e em contextos distintos são necessários para validar e aprimorar o modelo. A inclusão futura de marcadores de fragilidade pode enriquecer as análises. 39

É importante ressaltar que estimativas objetivas de risco podem orientar decisões compartilhadas médico-paciente, estratégias de vigilância e medidas sobre fatores modificáveis. A estimativa oferecida pela ferramenta aqui apresentada é especialmente confiável no contexto hospitalar no qual foi desenvolvida, sendo especialmente útil em hospitais especializados. Embora seu uso possa oferecer suporte a tomada de decisão em contextos de hospitais gerais, é importante ter em mente que o perfil dos pacientes pode mudar, de forma que a magnitude dos efeitos de risco pode ser imprecisa.

## Conclusão

Dislipidemia, sedentarismo, capacidade funcional reduzida (< 4 METs pelo DASI) e classificação ASA progressiva são fatores de risco isolados relevantes para eventos adversos até 30 dias após ATQ primária eletiva. Ressalta-se, portanto, a necessidade de uma avaliação pré-operatória ampliada, que considere não apenas a classificação ASA, mas também aspectos funcionais e comorbidades. A ferramenta de estratificação de risco proposta demonstrou acurácia satisfatória, representando um potencial recurso para a prática clínica. Contudo, para implementação da ferramenta são necessários estudos de validação externa em diferentes contextos assistenciais.
